# Hereditary Syphilis

**Published:** 1879-09

**Authors:** J. N. Hyde


### HEREDITARY SYPHILIS.

BY DR. J. N. HYDE.

VACCINO-SYPHILIS; OR, THE ACCIDENTAL TRANSMISSION OF SYPHILIS BY VACCINE LYMPH.—(*Med. Press and Circular*, Ap. 9, 1879, p. 279.)

Dr. Hugh Thomson's communication to the Glasgow Medico-Chirurgical Society commences with the promise of an interesting and valuable contribution to his subject, but continues and concludes with a disappointment. As the writer had never seen a case of vaccino-syphilis, his brief review of the literature of the subject is succeeded by an extended reference to the cases reported by Mr. Hutchinson and portrayed in the "Clinical Illustrations" of that author. Dr. Thomson, referring to the demonstration by Keber, of Dantzig, that "even clear vaccine lymph contains pus and blood corpuscles," suggests that to transmit syphilis from a vaccinifer, the presence in the lymph of visible blood globules is not requisite. The views of Seaton and DePaul are adduced in favor of the possibility of greater peril in vaccinations made after the exhaustion of the first and freshest lymph supply in the arm of any given vaccinifer, Thomson calling attention to the somewhat remarkable fact that in the Italian epidemics of syphilis, the same child was taxed for a supply of lymph sufficient to vaccinate fifty, sixty and even a hundred other children. The practical rules which the general consideration of the subject should suggest are: (*a.*) From a single vaccinifer, not more than ten or twelve persons should be vaccinated. (*b.*) The vaccinifer should, whenever possible, be more than four months old, that the period in which hereditary syphilis most commonly is displayed, may have elapsed. (*c.*) When in doubt regarding the immunity of the vaccinifer, always reject the lymph, not waiting for proof of syphilis.

Dr. Thomson concludes as follows: "I have said nothing of another method of avoiding syphilitic infection in vaccination, one which may be said to afford absolute security against it, viz., *animal vaccination*, or the employment of the calf or heifer only as vaccinifers. The reason is, that the drawbacks connected with such a mode of vaccinating are so great, though of a different sort, as so far to outweigh the small, it may be said infinitesimal, advantage it may have in respect to syphilis, that it is never likely to be adopted on a large scale in this country."

SYPHILIS BY VACCINATION WITH HUMAN VIRUS. — (*Gaz. des Hôpitaux — Tol. Med. and Surg. Jour.*, Mar., 1879, p. 125.)

Virus was taken from the seven-months-old child of a mother subsequently known to be syphilitic, the infant at the time presenting all the appearances of perfect health. With the virus thus obtained, twenty-five girls were vaccinated, twelve of whom in six weeks had ulcerations at the point of inoculation, succeeded by exanthem, oral and pharyngeal ulcers, anal condylomata, syphilitic ozæna, etc. Three others had suspicious lesions in the neighborhood of the vaccine sore, which were not followed by constitutional symptoms.

INFECTION OF A NURSE BY HER NURSLING. — (*Gazette Obstét.*, Ap. 5, 1879, p. 103.)

Dr. Guillery had under observation a woman with conjunctivitis, abscess near the lachrymal sac, inguinal adenopathy, and syphilitic "lichen" over the surface of the thighs and chest. When her own infant was fifteen months old, she concluded to nurse another child, and, being examined by a physician, was pronounced healthy.

The infant delivered to her charge was one month old, feeble and sickly in appearance, had a copious eruption over the thighs and legs, and fissures of the mouth and anus. It seized the breast with difficulty, on account of the habitual obstruction of the nasal fossæ. In fifteen days, the nurse found that she had fissure of the nipple. In two months the child died, but, towards the end of the first month, the nurse discovered a macular eruption upon the surface of the chest. Meantime she had not ceased to suckle her own child as well as the stranger; the former enjoyed health up to the time of the death of the latter, when the mother noticed that it displayed an exanthem over the body, and suffered from a conjunctivitis not unlike her own. These accidents on the part of the nurse's own child were eventually recognized as fortuitous; the infant was, in short, free from the disease.

The inferences Guillery seems to draw from these facts are certainly not those which an expert would consider warrantable. A simple fissure of a nurse's nipple coming in contact with the secreting lesions of the mouth of a syphilitic infant, and becoming thus the portal of infection, must of necessity be transformed into the primary manifestations of the infectious disease. Nor is it safe to decide that such transformation has not occurred because "chancres were not seen in that locality." Much less can it be thought possible that absorption of a virus could happen at the site of such fissures and the result be a species of so-called "syphilis d'emblée." Neither is it generally to be concluded that when papular syphilides exist upon the surface of the body, the most abundant crop will occur in the regions nearest to the site of inoculation. Such a manifestation must, in any case, be regarded as quite fortuitous.

ACQUIRED INFANTILE SYPHILIS. — ("De la Syphilis Infantile Acquise," par le Dr. Alfred Pontet; Paris, Delahaye, 1878.)

Pontet, in the consideration of his subject, devotes special attention to the questions regarding etiology and diagnosis. Under the title of "Accidental Syphilis," he considers the results of contact with the nipple and breast, vaccination, infection by the medium of toilet articles, and contagion where there is ignorance. Under the title of "Intentional Syphilis" are classed those cases which seem to result from a deplorable superstition, existing only in France, whereby it results that a female infant is infected directly from an adult. Syphilis may, however, be intentionally communicated from other motives.

In proof of the communication of syphilis, two circumstances require consideration before arriving at a diagnosis: 1st, the existence of an indurated chancre, it matters not in what region of the body; 2d, the traces of a traumatism, more or less violent, with or without rupture of the hymen. The author is inclined to believe that tertiary manifestations are rare as the result of acquired infantile syphilis, the disease in such cases attacking organs which are in the phase of evolution, and which with greater readiness admit of its modification or elimination.

HEREDITARY TRANSMISSION OF SYPHILIS. — (*Brit. Med. Jour.*, April 26th, 1879, p. 631.)

In Mr. Milton's case, a father was supposed cured before marriage, but had, nevertheless, the first-born dead; the second, a child healthy save for a slight eruption; the third, without any symptoms of disease; the fourth, distinctly syphilitic.

HEREDITARY BONE-SYPHILIS, MULTIPLE FRACTURES AND PSEUDO-PARALYSIS. — (*Cbl. f. Chir.*, No. 4, 1879; from *Bull. de la Soc. de Chir.*)

Polaillon reports the case of a woman, 21 years old, who had flat condylomata in the second month of pregnancy, and gave birth to a child at full term. This child had fracture of the left humerus and paresis of the right arm and lower extremities. The bones about the elbows and the entire left femur were thickened, and painful on pressure. There was no other external sign of syphilis. The child died in eight days of intestinal catarrh. *Post-mortem*, there was found fracture of the humerus and dislocation of the upper fragments without the formation of callus, though there was periosteal thickening. The right humerus was fractured at the same point without periosteal lesion and dislocation. Both fractures were respectively nine and eleven millimeters from the epiphyseal cartilage. The right femur was one-half centimeter longer than the left. The latter was of decidedly smaller volume in several places. There was a transverse fracture of the left femur without dislocation, about five centimeters below the epiphyseal cartilage. In the right femur there was a fracture near the upper end. Various enlargements were found on the other bones, with porotic condition in places. The fractures were considered as intra-uterine and not post-partum, in consequence of the evidences of inflammation.

